# MicroRNA‐Driven Regulation of β‐Cell Function in Type 2 Diabetes: Molecular Mechanisms, Network Insights, and Translational Perspectives

**DOI:** 10.1111/apha.70288

**Published:** 2026-08-07

**Authors:** Lena Eliasson, Alexandros Karagiannopoulos, Mototsugu Nagao, Anna Wendt

**Affiliations:** ^1^ Unit of Islet Cell Exocytosis, Lund University Diabetes Centre (LUDC), Department of Clinical Sciences Malmö Lund University Lund Sweden; ^2^ Clinical Research Centre (CRC) Skåne University Hospital (SUS) Malmö Sweden; ^3^ Science for Life Laboratory (SciLifeLab) Lund University Lund Sweden; ^4^ Bioinformatics Unit, Lund University Diabetes Centre (LUDC), Department of Clinical Sciences Malmö Lund University Lund Sweden; ^5^ Division of Diabetology and Metabolism, Department of Internal Medicine Tokyo Women's Medical University School of Medicine Tokyo Japan

**Keywords:** alpha cell, beta cell, diabetes, exocytosis, human islet, insulin secretion, microRNA

## Abstract

MicroRNAs (miRNAs) have emerged as central regulators of pancreatic islet biology, influencing β‐cell development, proliferation, and function. In type 2 diabetes (T2D), both adaptive and maladaptive miRNA responses shape β‐cell compensation and progressive secretory dysfunction. Here, we review current insights into the regulation of insulin secretion, with a focus on exocytosis and the autocrine and paracrine regulation of β‐cells, and discuss the involvement of miRNAs in these processes. We describe miRNA biogenesis and the regulation of miRNAs in β‐cells, focusing on glucose‐ and cAMP‐responsive miRNAs and epigenetic control. We summarize mechanistic evidence linking individual miRNAs to the regulation of β‐cell function during T2D development, including effects on metabolic signaling and the exocytotic machinery, and compare results from humans, rodents, and cell lines, while emphasizing both the need for and difficulties of performing these investigations in β‐cells from human donors. Moreover, recent network‐level analyses reveal that T2D is characterized not by isolated miRNA changes but by coordinated changes in miRNA–mRNA regulatory pathways that converge on reduced and/or compensatory insulin secretion. Beyond cell‐intrinsic actions, we discuss how circulating miRNAs transported via extracellular vesicles (EVs) mediate crosstalk between β‐cells and peripheral tissues, influencing insulin resistance, β‐cell function, and systemic glucose homeostasis. Finally, we highlight the potential of circulating miRNAs as diagnostic and predictive biomarkers, as well as the use of EVs and miRNAs for therapeutic applications, while underscoring the technical challenges that currently limit clinical translation. Nevertheless, advances in technical, network, and inter‐organ analyses position miRNAs as promising biomarkers and therapeutic targets in T2D.

## Practioner Points

- MicroRNAs modulate β‐cell compensation, dysfunction, and progression of type 2 diabetes.
- Defects in β‐cell exocytotic machinery contribute to impaired insulin secretion in type 2 diabetes.
- MicroRNAs and extracellular vesicles represent promising biomarkers and therapeutic targets in type 2 diabetes.

## 1 Introduction

Diabetes is a chronic metabolic disorder marked by impaired regulation of blood glucose levels. Almost 600 million adults currently live with diabetes worldwide [1] and the majority (approximately 90%) are classified as having type 2 diabetes (T2D). The projected increase to ~800 million in 2050, together with the absence of curative therapies, has made T2D a major global health challenge.

**FIGURE 1:**
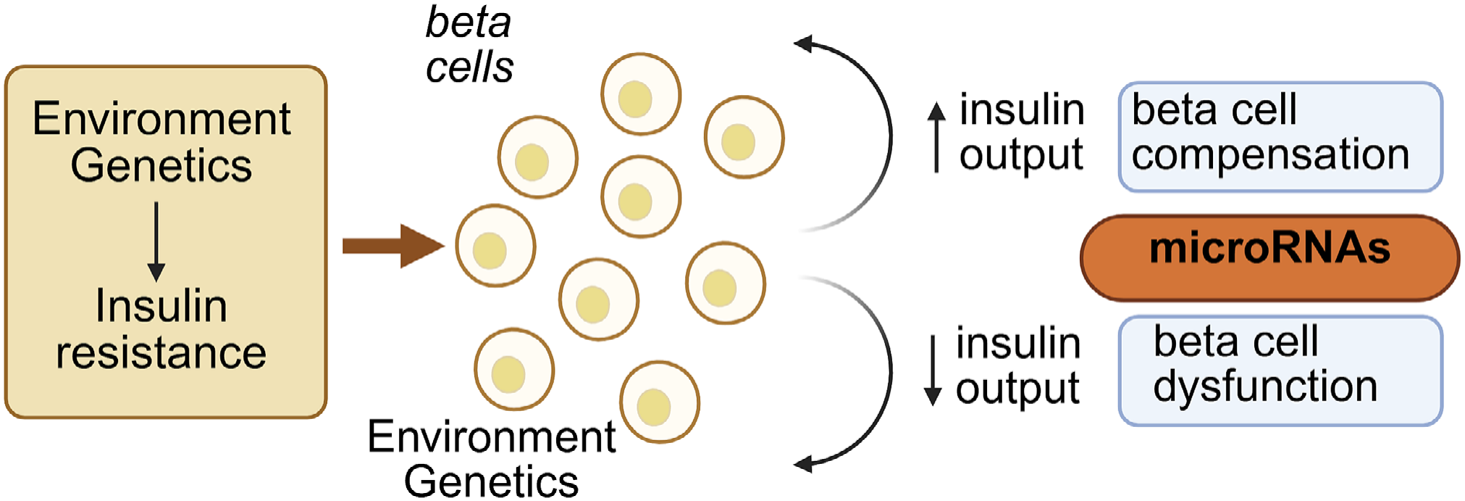
β‐cell compensation and dysfunction in the pathogenesis of T2D. During the development of T2D, environmental and genetic factors influence pancreatic β‐cells directly and indirectly through the development of insulin resistance. In response to increased metabolic demand, β‐cells initially undergo compensatory changes, resulting in increased insulin secretion. However, sustained stress or susceptibility factors can lead to β‐cell dysfunction, characterized by reduced insulin output. Importantly, β‐cell dysfunction may also arise independently of insulin resistance, reflecting direct effects of environmental and genetic factors on β‐cell function. MicroRNAs (miRNAs) are proposed to act as key regulators of these adaptive and maladaptive processes, in which some miRNA expression changes are part of the etiology of T2D, whereas others occur as a compensatory mechanism against insulin resistance. Created in BioRender. Eliasson, L. (2026) https://BioRender.com/i7ux3n4.

T2D is a multifactorial disease caused by a relative deficiency in insulin secretion from pancreatic β‐cells combined with insulin resistance in target tissues. The contribution of these two mechanisms varies among people and across the development of T2D. Impaired insulin secretion reflects two different but related processes (Figure 1): β‐cell compensation and β‐cell dysfunction [2, 3]. To meet the higher metabolic demand associated with insulin resistance, β‐cells increase their insulin output, a phase referred to as β‐cell compensation. Failure of this compensatory response and the resulting insufficient insulin release contributes to the onset of T2D. In parallel, β‐cell dysfunction occurs, that is, defects in insulin secretion can be detected even before clinical diagnosis, including loss of first‐phase insulin release and a reduction in second‐phase insulin secretion [4].

β‐cells are, together with α‐cells and a few other pancreatic cell types, organized into highly specialized micro‐organs known as the islets of Langerhans (islets). An adult human individual has approximately one to two million islets dispersed across the pancreas and, collectively, these islets are referred to as the endocrine part of the pancreas. In addition to insulin secretory defects, people with T2D display dysregulated glucagon secretion from the pancreatic α‐cells, suggesting that T2D can be viewed as a bi‐hormonal disease with dysregulation of both insulin and glucagon signaling [5]. The secretion of both insulin and glucagon is tightly regulated. Our understanding of β‐cell regulation is much clearer than that of α‐cells, but both cell types are responsive to nutritional status, with the response fine‐tuned by an integrated network of hormonal, neuronal, and paracrine signals.

In addition to well‐known regulators of insulin secretion such as glucose and cAMP [6], recent studies have put forward microRNAs (miRNAs) as important fine‐tuning controllers of islet hormone secretion (see e.g., [2, 3, 7, 8, 9]). MiRNAs are small non‐coding RNAs that regulate post‐transcriptional expression of specific target proteins by binding to sites primarily located in the 3′ untranslated region (3′ UTR) of their complementary messenger RNAs. During the development of T2D, miRNAs play important roles within the β‐cells where altered expression of some miRNAs supports compensatory enhanced insulin‐secretory responses to insulin resistance, whereas dysregulation of others contributes to β‐cell dysfunction and reduced insulin secretion.

**FIGURE 2:**
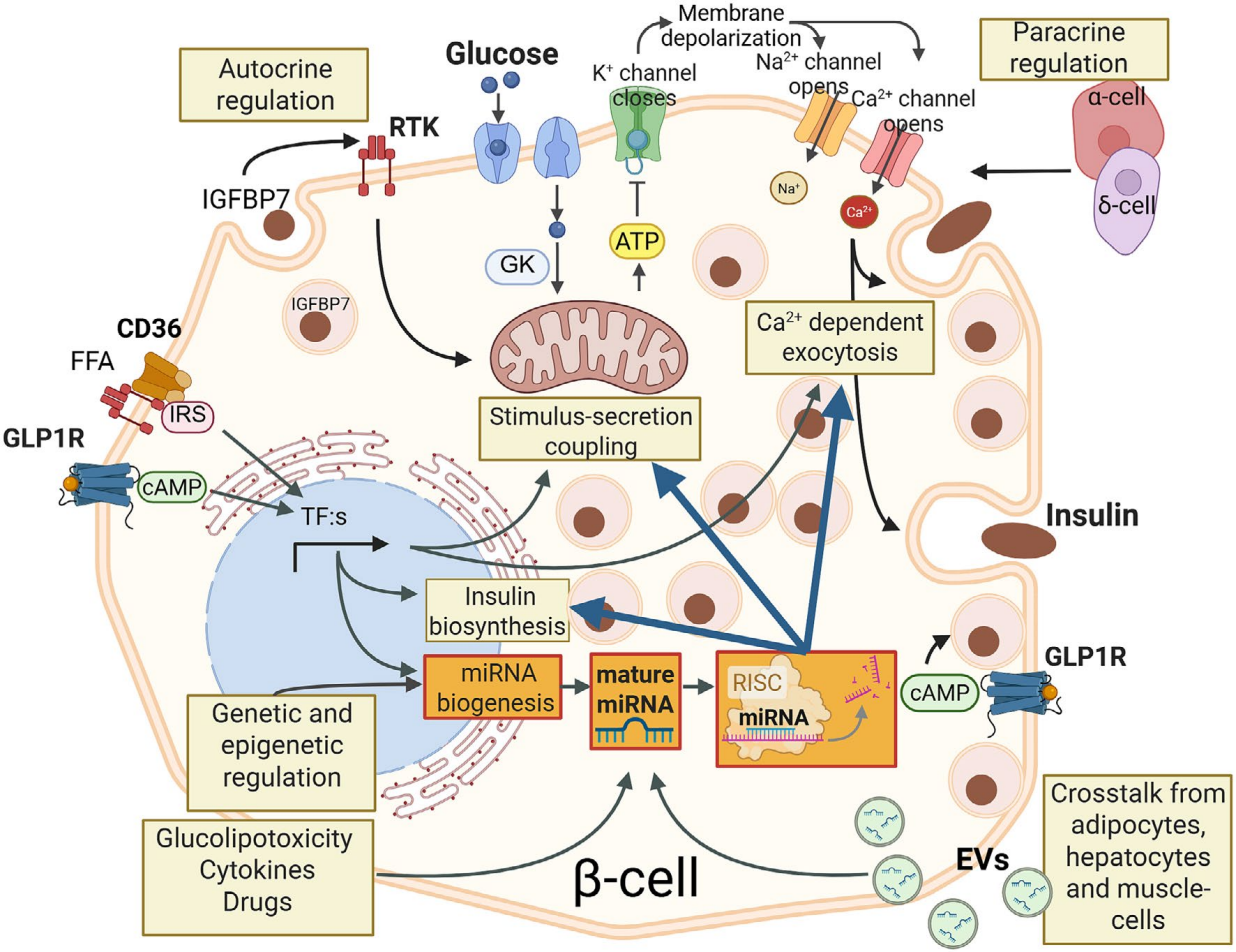
Model of miRNA‐mediated regulation of β‐cell function. Schematic overview of miRNA biogenesis, regulation, and function in the β‐cell. miRNAs are generated through miRNA biogenesis, giving rise to mature miRNAs that are incorporated into the RNA‐induced silencing complex (RISC) complex to regulate target gene expression. This process is influenced by genetic and epigenetic regulation as well as environmental factors including glucolipotoxicity, cytokines, and drugs. In addition to intracellular biogenesis, miRNAs can be delivered via extracellular vesicles (EVs), reflecting crosstalk from adipocytes, hepatocytes, and muscle cells. Within the β‐cell, miRNAs regulate key processes including insulin biosynthesis, stimulus‐secretion coupling, and Ca^2+^‐dependent exocytosis. Glucose is the main regulator of insulin secretion. The stimulus‐secretion coupling of the β‐cell starts with the uptake of glucose through glucose transporters, through glucokinase (GK) activity and mitochondrial metabolism, ATP levels increase, leading to closure of ATP‐dependent K^+^ channels, opening of Na^+^‐ and Ca^2+^‐channels, and triggering of Ca^2+^ influx and insulin secretion. Incretin signaling through GLP1R increases cAMP, activates transcription factors (TF:S), and modulates both insulin secretion and miRNA expression. TFs can also be modulated via other mechanisms such as the free fatty acid (FFA) transporter CD36 and the insulin signaling pathway. Autocrine regulation (here exemplified by IGFBP7 released from insulin granules regulating the β‐cell most likely via receptor tyrosine kinase (RTK)) and paracrine regulation from α‐cells and δ‐cells further fine‐tune β‐cell activity. Together, this model illustrates how miRNAs integrate intracellular signaling pathways, intercellular communication, and metabolic inputs to regulate β‐cell function and insulin secretion. Created in BioRender. Eliasson, L. (2026) https://BioRender.com/i7ux3n4.

Our understanding of the potential of miRNAs has expanded considerably since the discovery of the first vertebrate miRNAs [10, 11]. In this review, we summarize recent advances in the role of miRNAs in T2D, with a particular focus on pancreatic β‐cells and the regulation of insulin secretion (Figure 2).

## 2 Mechanistic Regulation of Insulin Secretion

Insulin is the only known glucose‐lowering hormone. It is secreted from the pancreatic β‐cells in the postprandial state to enable key metabolic tissues, including the liver, adipose tissue, and skeletal muscle, to take up, utilize, and store excess nutrients. Insulin release is tightly regulated in several steps including insulin biosynthesis, cellular metabolism, ion‐channel activity, and the Ca^2+^‐dependent exocytotic process. In T2D, these different processes become impaired due to both physiological defects and changes in the expression of key molecules. The latter is driven by genetic and/or epigenetic mechanisms, including the involvement of non‐coding RNAs such as miRNAs (Figure 2). In this section, we outline the fundamental principles of insulin secretion.

### 2.1 Core Mechanisms Underlying β‐Cell Insulin Secretion

#### 2.1.1 Glucose‐Driven Stimulus‐Secretion Coupling in β‐Cells

Glucose is the main trigger for insulin release, and the mechanism is described in the well‐established model of the stimulus‐secretion coupling of the β‐cell [6]. In short, insulin secretion is triggered by glucose entering the β‐cell via facilitated diffusion mainly through the insulin‐independent glucose transporters GLUT1 and GLUT2. Once inside, glucose is metabolized to generate ATP at the expense of ADP. The resulting increase in intracellular ATP/ADP ratio closes K_ATP_ channels, allowing for cell‐membrane depolarization. This opens voltage‐gated Ca^2+^ channels, resulting in an influx of Ca^2+^ and subsequent insulin release. Several factors have been identified that can amplify glucose‐induced insulin secretion, most notably cAMP, which is increased by several mechanisms including hormonal signaling by the gut hormones GLP‐1 and GIP in both rodents and humans [12, 13, 14].

#### 2.1.2 Insulin Biosynthesis, Processing, and Granule Formation

Transcription of the human insulin gene (*INS*), on chromosome 11, is tightly regulated by glucose and is controlled by several transcription factors including PDX1 and MAFA. The mRNA is then processed and translated into preproinsulin, which undergoes sequential processing to proinsulin and ultimately to mature insulin. During this process, proinsulin is packaged into large dense‐core secretory granules. Its conversion to mature insulin requires endopeptidase‐mediated cleavage into insulin and C‐peptide, a reaction that is dependent on an acidic intragranular environment [15]. Impaired processing leads to increased release of proinsulin, as can be observed, for example, in people with Cystic Fibrosis Related Diabetes (CFRD) [16].

#### 2.1.3 Exocytosis and Its Dysfunction in T2D

Insulin is released from the β‐cell through a complex exocytotic process. Defects in the exocytotic machinery contribute to T2D at multiple stages of the secretory cascade. For example, exocytosis is normally amplified by GIP and GLP‐1 through increases in intracellular cAMP [13], an effect that is impaired in T2D [17]. Extensive research over many years, initially in rodents and subsequently in human β‐cells, has established that insulin granules must translocate to the plasma membrane, undergo Ca^2+^‐, ATP‐, and cAMP‐dependent priming, dock via specific proteins, and ultimately proceed through Ca^2+^‐dependent membrane fusion [14]. Beyond regulating granule recruitment and priming, cAMP signaling modulates fusion pore expansion through Epac2, thereby influencing the balance between hormone and transmitter release during exocytosis [18]. Moreover, high‐resolution biophysical studies [19] indicate that granule fusion itself is compromised and that β‐cells from people with T2D exhibit impaired fusion‐pore expansion and premature pore closure, limiting cargo discharge, despite normal upstream Ca^2+^ signaling.

The loss of first‐phase insulin secretion in human T2D has also been linked to defective spatial coupling between voltage‐sensitive Ca^2+^ channels and insulin granules, a process that normally enables rapid and efficient exocytosis. To generate the localized high Ca^2+^ microdomains required for this process, studies in human islets and INS‐1 cells have identified Munc13 as a key mediator [20]. Disruption of this channel‐granule coupling abolishes the pool of primed granules, reduces granule‐localized Ca^2+^ influx, and impairs the tight temporal coupling between depolarization and exocytosis. Work from Echeverry et al. has further shown that insulin granules recruit approximately six copies of Munc13 during priming, and that Munc13 regulates granule release probability through two distinct C2B‐dependent mechanisms controlling its recruitment to and activation at the release site [21]. Mechanistically, these defects likely reflect impaired Ca^2+^ channel recruitment during granule priming and reduced Munc13 association, highlighting altered release site organization as a key driver of β‐cell dysfunction in human T2D.

Moreover, the expression of several exocytotic genes is reduced in human islets from people with T2D [22], including the calcium‐insensitive synaptotagmins, *SYT11* and *SYT13*. Recent findings published in *Acta Physiologica* [23] showed that these proteins localize to insulin granules and modulate secretion, with *SYT11* knockdown enhancing and *SYT13* knockdown reducing glucose‐ and K^+^‐stimulated insulin release in the rodent insulin‐secreting cell line INS‐1832/13. Thus, SYT11 and SYT13 proteins are both important regulators of β‐cell exocytosis whose altered expression may contribute to T2D‐related secretory dysfunction. Transcriptional dysregulation also contributes to exocytotic failure. Expression of the transcription factor genes *MAFA* and *MAFB* is reduced in islets from people with T2D, resulting in lowered levels of essential exocytotic genes such as *STX1A, STXBP1, and SYT7*, and thus weaker granule‐fusion capacity [24]. Downregulation of exocytosis‐related genes and impaired exocytosis have also been linked to obesity, as demonstrated in INS‐1E cells overexpressing the long‐chain fatty acid transporter CD36. Consistent with this, CD36 protein expression is elevated in islets from human donors with obesity and T2D [25], further supporting a link between dysregulated exocytosis and the pathogenesis of T2D.

An additional mechanism recently described [26] involves Syntaxin‐2 (STX2), which competes with Syntaxin‐1A at granule‐docking sites and thereby functions as an inhibitory SNARE. In β‐cells from T2D donors, excessive STX2 recruitment to plasma‐membrane microdomains suppresses trans‐SNARE‐complex assembly, while glucose‐induced STX2 membrane flipping relieves this inhibition. Defects in the flipping mechanism may therefore contribute to secretory failure in T2D.

Taken together, these findings underscore that restoring insulin secretion in T2D critically depends on re‐establishing efficient exocytosis, requiring interventions that target the entire exocytotic axis, from cytoskeletal remodeling to SNARE regulation, granule priming, and membrane fusion. In this context, miRNAs may represent a potentially useful class of regulators, given that a single miRNA can modulate multiple genes and pathways at the same time. Indeed, the knowledge that both first‐ and second‐phase insulin secretion are associated with differentially expressed miRNAs in islets from people with T2D [27], together with data showing the capacity of individual miRNAs to modulate multiple exocytosis‐related genes [2, 27], and the recent links between circulating miRNAs and β‐cell function [28] support a potential role for miRNAs as higher‐level regulators of β‐cell exocytosis. Hence, highlighting the need for further investigation of their functional and therapeutic relevance.

### 2.2 Autocrine and Paracrine Hormonal Regulation Within the Islets

Several local paracrine/autocrine factors modulate insulin secretion (Figure 2). For more details, see, for example, reviews by Huising [29] and Tröster et al. [30]. In short, it has been known for ~50 years that somatostatin released from islet δ‐cells negatively modulates both insulin and glucagon secretion, thereby preventing excessive hormone output as glucose levels normalize. Beyond their counter‐regulatory role, α‐cells release glucagon, GLP‐1, acetylcholine, and CRH, which enhance β‐cell insulin secretion during hyperglycaemia by increasing cAMP and promoting granule recruitment. These stimulatory and inhibitory signals operate alongside additional β‐cell–derived factors, such as serotonin and GABA, that modulate α‐cell activity and contribute to the characteristic pulsatile and phase‐shifted pattern of islet hormone secretion. Recently insulin‐like growth factor binding protein 7 (IGFBP7) has been identified as a novel regulator of β‐cell function [31]. IGFBP7 is upregulated in islets (both α‐ and β‐cells) from people with T2D and impairs glucose‐stimulated insulin secretion, and intracellular inhibition enhances insulin secretion and reduces IGFBP7 release in the human β‐cell line EndoC‐βH1. These findings suggest an autocrine role in β‐cells.

The importance of the intra‐islet network has lately been emphasized, and it has become clear that insulin secretion from the β‐cells and glucagon secretion from the α‐cells reflect the net balance of nutrient input and paracrine amplification and inhibition [29]. Shifts in this balance contribute directly to the dysregulated hormone secretion seen in diabetes, underscoring that the disease reflects disrupted intra‐islet communication as much as impaired α‐ and β‐cell function. For instance, glucagon secretion is abnormal in both type 1 diabetes (T1D) and T2D, and the underlying mechanism(s) responsible for this are under investigation. It has long been acknowledged that glucagon secretion is under strict paracrine and hormonal control and, recently, the importance of somatostatin‐induced inhibition of glucagon secretion has been revisited. In high‐fat‐fed mice, somatostatin secretion, as well as somatostatin sensitivity, is reduced in the α‐cells with effects on the glucagon secretory machinery, suggesting a role for dysregulated somatostatin release in pre‐diabetes [32]. In contrast, in human islets from people with T1D, somatostatin release is markedly increased due to reduced electrical coupling between β‐ and δ‐cells [33] possibly explaining why hypoglycaemia‐induced glucagon secretion is impaired in these individuals.

By contrast, Perez‐Frances et al. [34] demonstrated that mice and human organoids composed exclusively of β‐cells can maintain, and in some contexts even enhance, glucose‐stimulated insulin secretion and metabolic control compared with intact islets, indicating that β‐cells possess a strong intrinsic capacity for glucose homeostasis independent of paracrine input from other endocrine cell types. These findings challenge the long‐held assumption that α‐ and δ‐cell support is indispensable and suggest practical advantages for engineering β‐cell–only tissues for therapeutic applications. However, this is an artificial setting, and it is more likely that paracrine regulation between neighboring cells in the islet plays essential roles in β‐cell function.

Overall, these data support a model in which islet hormone secretion arises from the dynamic, reciprocal interactions among α‐, β‐, and δ‐cells, while highlighting that the relevance and extent of this paracrine coordination vary with physiological and experimental context. In addition, emerging evidence suggests that extracellular vesicles (EVs) may represent an additional layer of intra‐islet communication, a concept explored further below.

## 3 Regulation of miRNAs in Pancreatic β‐Cells

Victor Ambros and Gary Ruvkun were awarded the 2024 Nobel Prize in Physiology or Medicine for their discovery and characterization of miRNAs. Their early 1990s research in *C. elegans* , investigating lin‐4 and lin‐14, revealed a previously unknown principle of gene regulation [10, 35]. The first miRNA identified in pancreatic β‐cells, miR‐375, was discovered a few years later [11]. Since then, hundreds of miRNAs have been identified, and our understanding of their functions has expanded considerably. However, the full complexity of miRNA‐mediated networks in β‐cells is only beginning to emerge, leaving many mechanistic and translational questions open for investigation.

### 3.1 Canonical and Non‐Canonical miRNA Biogenesis

MiRNAs are short (~22 nt) RNA‐molecules [36] that, unlike mRNA, are non‐coding and instead regulate protein expression by post‐transcriptional regulation of their target mRNAs. As a general rule, miRNAs repress protein expression although there are examples of increased protein expression instead [37]. Genetically, miRNAs are located within protein‐coding genes, primarily in the intronic regions and less frequently in exons. Research has also shown that a subset of miRNAs is independently transcribed and regulated by their own promoter elements [38].

MiRNAs are typically transcribed as pri‐miRNAs with their characteristic hairpin structures [39] (Figure 2). The pri‐miRNA is processed into a pre‐miRNA by the RNase III enzyme DROSHA, in complex with DGCR8, and is subsequently exported into the cytoplasm of the cell by Exportin5. In the cytoplasm, the endonuclease DICER processes pre‐miRNA into a mature miRNA duplex. Both the 3p (originating from the 3′ end of the pri‐miRNA hairpin) and the 5p (originating from the 5′ end of the pri‐miRNA hairpin) arms can be biologically active. The miRNA duplex is loaded into the Argonaute protein (AGO), forming the RNA‐induced silencing complex (RISC), in which one of the strands is discarded, and only the “guide” strand is retained. In RISC, the miRNA guide strand directs binding to target mRNAs primarily via its seed sequence (nucleotides 2–7 or 2–8), which forms perfect complementarity with the corresponding region in the 3′ UTR of the mRNA. This causes post‐transcriptional repression through mRNA cleavage, translational repression, and/or mRNA destabilization. MiRNAs sharing an identical seed sequence are assigned to the same family [40, 41]. Such family members generally have related evolutionary origins and tend to carry out similar functions despite differences in their overall sequence or structure.

MiRNA regulation is far more complex than the canonical miRNA biogenesis pathway described above indicates. This is demonstrated by the following: First, one miRNA can regulate many mRNAs and, conversely, one mRNA can be regulated by several miRNAs [42, 43]. This is most likely because miRNA‐mRNA interactions do not require full complementarity across the full length of the miRNA. Second, although miRNA binding is classically associated with the 3′ UTR, growing evidence shows that target sites can also be found within protein‐coding sequences [44]. Recent data further demonstrate that miRNAs can bind and repress targets even without perfect seed‐sequence pairing [45]. Third, broad post‐transcriptional regulation of miRNA transcripts is mediated by specific RNA‐binding proteins (RBPs) [39]. These proteins influence multiple steps of miRNA maturation, including precursor processing and the loading of mature miRNAs into the RISC complex. In addition, RBPs integrate different RNA‐processing pathways by facilitating their crosstalk, coordinating aspects of miRNA biogenesis during transcription and contributing to miRNA sorting into EVs. Fourth, miRNA regulation is functionally compartmentalized and dynamically modulated by the abundance of miRNAs, mRNAs, RBPs, and other associated molecules [46]. Finally, the biogenesis of miRNAs is highly flexible rather than strictly linear, and several molecular mechanisms can shape their maturation. Post‐transcriptional events, such as imprecise cleavage by DROSHA or DICER, RNA editing, and more, all contribute to the production of sequence variants known as isomiRs [47]. In human islets and β‐cells [48], isomiRs constitute a substantial proportion of the miRNA landscape (40%–60% of total miRNA reads), highlighting that they are integral components of the regulatory network rather than rare byproducts. By altering the seed sequence or affecting miRNA stability, isomiRs can redirect target specificity and regulate gene sets distinct from their canonical counterparts. Functionally, Auddino et al. have identified β‐cell–enriched isomiRs such as isomiR‐411‐5p‐Ext5p(+1) [48], which uniquely modulate insulin secretion and vesicle‐trafficking pathways.

### 3.2 Regulation of miRNA Expression in β‐Cells

Environmental signals, including blood glucose levels, metabolic stress, and pro‐inflammatory cytokines, have been shown to fine‐tune miRNA expression, allowing β‐cells to rapidly adapt to changing physiological demands (Figure 2). This regulation involves mechanisms such as transcriptional control of miRNA expression together with epigenetic mechanisms that modulate chromatin accessibility at miRNA gene loci. In diabetes, chronic metabolic and inflammatory perturbations reshape the β‐cell miRNA landscape, contributing to dysfunction and disease progression.

Short‐term and long‐term glucose responsiveness of miRNAs has been demonstrated in several experimental models. In the Goto Kakizaki (GK) rat, expression of miR‐130a and miR‐212 increased already after 1 h at 16.7 mM glucose, illustrating short‐term glucose‐dependent induction of miRNA expression [49]. A similar pattern has been reported in humans, where circulating miRNA levels changed in response to glucose during an oral glucose tolerance test (OGTT) [50], suggesting acute glucose‐sensitive miRNA regulation in vivo.

Over longer exposures (24–72 h), numerous studies have identified miRNAs that respond to chronic high glucose or to combined glucose and palmitate treatment, the latter reflecting glucolipotoxic conditions typical of T2D [2]. For example, Cheung et al. reported AMPK‐dependent upregulation of miR‐125b in response to increasing glucose concentrations in both mouse and human islets [51] and a positive correlation beween BMI and miR‐125b expression, suggesting a potential association with obesity. Also, Wang et al. highlighted obesity‐induced alterations in miRNA expression, including increased levels of miR‐146b [52]. Cheung et al. further showed that miR‐125b is implicated in the regulation of targets such as the lysosomal hydrolase transporter *M6pr* and the mitochondrial fission regulator *Mtfp1*, supporting a possible role in the control of organelle dynamics in β‐cells.

Several other miRNAs have been reported to exhibit increased expression in islets from human donors or rodent models under conditions of elevated glucose and/or glucolipotoxicity, as well as in islets from obese rodent models. These include miR‐34a (GK rat, human) [53, 54], miR‐199 (mouse, human) [54, 55], miR‐212 (human) [56] and miR‐483‐5p (high‐fat‐diet mouse model) [57]. In contrast, miR‐299 and miR‐204 have been shown to be downregulated during a 48 h glucolipotoxicity treatment [54]. Taken together, these findings support a role for obesity‐associated and glucolipotoxicity‐regulated miRNAs in islet biology.

The miR‐29 family has emerged as an important regulator of β‐cell function, with effects on mitochondrial metabolism, insulin secretion, proliferation, and intercellular signaling [58, 59]. However, its regulation in β‐cells is not uniform across experimental systems. Instead, available data indicate that miR‐29 expression is highly context‐dependent, varying with species, diabetic models, β‐cell lines, the duration and type of metabolic exposure, and disease stage. This complexity is particularly evident when comparing insulin‐secreting cell lines, rodent islets, and human islets from people with T2D. In the INS‐1 832/13 β‐cell line, elevated glucose downregulates miR‐29a, miR‐29b, and miR‐29c, an effect also observed in islets from db/db mice [59]. This reduction is associated with enhanced insulin secretion and mitochondrial activity, suggesting that decreased miR‐29 may support compensatory β‐cell function in metabolically challenged states. In contrast, miR‐29 expression is increased in islets from people with T2D and in GK rat islets [59], consistent with a role in β‐cell dysfunction under chronic diabetic conditions. Importantly, contrasting findings have also been reported, including glucose‐induced upregulation of miR‐29 in INS‐1E cells and primary islets [58], underscoring the influence of experimental conditions such as exposure time and glucose levels.

Mechanistic studies have further shown that GLP‐1 induces upregulation of miR‐132 and miR‐212 in β‐cells via a cAMP/PKA‐dependent pathway in insulin‐secreting cell lines as well as rodent and human islets [60], where these miRNAs enhance glucose‐stimulated insulin secretion and contribute to β‐cell function. At the transcriptional level, their expression is regulated by factors including CRTC1 [61], Camta1/2, and Nkx2‐2 [62]. Notably, this regulatory axis is impaired in GK rat islets, where reduced expression of these transcriptional regulators is associated with diminished miR‐212/132 expression and β‐cell dysfunction. In addition, the commonly used antidiabetic drug metformin has been shown to increase the levels of miR‐184 and miR‐125b via *AMPK* and *LKB1* [63]. Finally, miRNA expression can also be modulated by epigenetic mechanisms such as DNA methylation [64, 65].

### 3.3 Network‐Level miRNA Regulation in Human Islets

Because individual miRNAs can target many different genes and many protein‐coding genes are regulated by multiple miRNAs, these RNA molecules collectively regulate a wide range of cellular processes. Consequently, many pathways are shaped by the combined actions of multiple miRNAs. The importance of this collective regulatory network is underscored by β‐cell–specific Dicer deletion in mice [66, 67], which disrupts miRNA processing and results in impaired insulin secretion and reduced β‐cell mass.

In a recent study from our group [27], we performed a comprehensive network‐level analysis of miRNA regulation in human islets from people with T2D compared with those without diabetes, identifying 70 differentially expressed miRNAs, of which 34 were upregulated and 36 downregulated in islets from people with T2D. The integrative miRNA‐mRNA network analysis revealed 4951 negatively associated mRNA targets, with upregulated miRNAs accounting for nearly twice as many targets as downregulated ones, emphasizing their stronger contribution to T2D‐related regulatory changes. Co‐expression clustering further demonstrated that only the mRNA targets of upregulated miRNAs were enriched in pathways directly related to insulin secretion, highlighting a coordinated network affecting β‐cell stimulus–secretion coupling. Within this network, miR‐101‐3p emerged as a central regulator, having the highest number of mRNA targets and influencing both phases of glucose‐stimulated insulin secretion. We propose that miR‐101‐3p acts as a compensatory miRNA, elevating insulin secretion in T2D. Indeed, overexpression of miR‐101‐3p in human EndoC‐βH1 cells increased glucose‐induced insulin secretion and repressed both mRNA and protein expression of the target Cell Adhesion Molecule 1 (CADM1) [27], recently shown to affect insulin secretion through remodeling of the actin cytoskeleton and interaction with exocytotic proteins [68].

Our findings complement other large‐scale genetic analyses of human islets, such as the studies by Taylor et al. [69] and Auddino et al. [48]. Taylor and co‐workers identified heritable islet miRNAs, miRNA‐eQTLs, and a smaller set of miRNAs associated with T2D status and glycaemic traits, whereas Auddino and co‐workers mainly focused on isomiRs. Notably, comparison of dysregulated miRNAs across studies [27], including those by Taylor et al. and Cowan et al., reveals limited overlap. Only miR‐187‐3p is consistently identified as upregulated, while miR‐9‐5p and miR‐30a‐5p show partial overlap. These observations underscore the importance of harmonizing study design and experimental setup for large‐scale small RNA sequencing, including the quality of human islets used. Regardless of differences between studies, current network‐level analyses indicate that T2D is characterized not by isolated miRNA changes but by broader rewiring of miRNA–mRNA regulatory networks, with convergence on pathways controlling insulin secretion despite heterogeneity at the level of individual miRNAs across human cohorts. Further evaluation is needed to fully understand how miRNA‐regulated networks fine‐tune β‐cell regulation and whether specific miRNAs are key nodes within this network.

### 3.4 MiRNA‐Mediated Regulation of Insulin Secretion in Human Islets

With respect to insulin secretion, miRNAs regulate target genes involved in multiple stages of the secretory pathway, including insulin gene transcription, insulin biosynthesis and storage, and all major components of the canonical stimulus–secretion coupling cascade (Figure 2). This includes mitochondrial metabolism and ATP production, ion channel activity, and the final steps of vesicle trafficking and exocytosis.

In line with these findings, several studies have reported miRNAs to be differentially expressed in islets from people with T2D, including members of the miR‐200 and miR‐7 families [3]. The miR‐7 family regulates β‐cell development and exocytosis, and its reduced expression in T2D may represent a compensatory mechanism to support insulin secretion [70]. The miR‐200 family controls β‐cell secretion as well as survival. Expression of miR‐200c is increased in islets from people with T2D. Data from both human islets and the human EndoC‐βH1 cells show that elevated miR‐200c reduces insulin secretion by targeting ETV5, thereby impairing β‐cell exocytosis [71]. In addition, the miR‐200 family has been identified as a key regulator of β‐cell survival and identity based on studies in mouse models and cell lines. Mechanistically, miR‐200 targets mRNAs associated with protective pathways, including *Zeb1*, *Dnajc3*, *Xiap*, and potentially *Jazf1*, and activates pro‐apoptotic signaling via Trp53/Bax [72], while upstream factors such as TXNIP further drive miR‐200 expression [73]. The miR‐200–Zeb1 axis also influences epithelial‐mesenchymal transition and β‐cell identity, linking apoptosis, dedifferentiation, and stress responses [74]. Interestingly, differential expression of *JAZF1* is confirmed in islets from people with T2D [71]. Together, these miRNAs represent important contributors to β‐cell dysfunction and promising therapeutic targets for improving insulin secretion in T2D.

**FIGURE 3:**
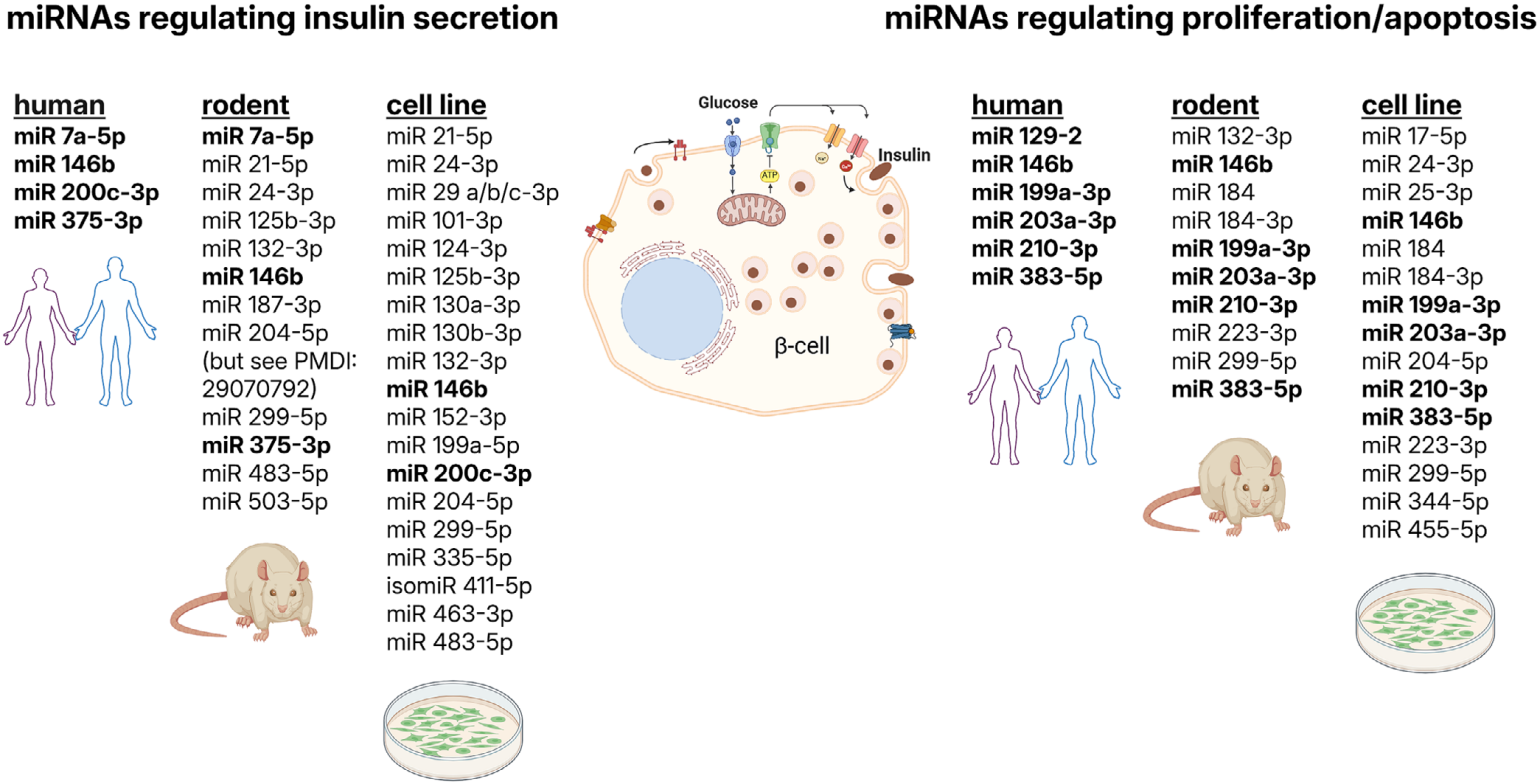
MicroRNAs regulating β‐cell function, survival, and proliferation in T2D. In T2D, insulin secretion as well as β‐cell apoptosis and proliferative capacity are dysregulated. This figure summarizes miRNAs that are associated with human T2D (often differentially expressed) and have been experimentally shown to regulate insulin secretion or β‐cell apoptosis/proliferation in human primary cells (human), rodent primary cells (rodent), or β‐cell lines (cell line). MiRNAs shown in bold have been experimentally validated in primary human islets or β‐cells. Data are from Karagiannopoulos et al. [3], Cowan et al. [27, 59], D'Addio et al. [75], Yuan et al. [57], Zhou et al. [76], Auddino et al. [48], Wang et al. [52]. Created in BioRender. Eliasson, L. Wendt, A (2026) https://BioRender.com/i7ux3n4.

Two miRNAs from the miR‐7 and miR‐200 families, miR‐7a‐5p and miR‐200c‐3p, stand out as central regulators of glucose‐stimulated insulin secretion, with functional data available from human islets and/or β‐cells. This is also the case for miR‐146b and miR‐375‐3p (Figure 3). Hence, of the 23 miRNAs reported as differentially expressed in islets from people with T2D across different studies (Figure 3), only 4 are functionally validated as mechanistic regulators of glucose‐stimulated insulin secretion in human islets. Likewise, only 6 of the 19 miRNAs differentially expressed in islets from people with T2D have been functionally validated in human islets to regulate β‐cell proliferation and apoptosis. Instead, functional follow‐up studies are often performed in cell lines or rodent models. This is due to the limited availability of human islets, particularly from donors with T2D. Therefore, studies in animal models with diabetes, as well as in models with genetic deletion or overexpression of specific miRNAs, have demonstrated mechanistic perturbation of miRNA‐regulated pathways affecting β‐cell function. The combination of human islet data and animal models can be exemplified by recent work by Zhou et al., who showed that miR‐503‐5p is upregulated in human β‐cells during inflammation and diabetes [76], whereas mechanistic experiments were performed in mice lacking the miR‐503 cluster and in MIN6 cells. It is often necessary to integrate functional data from cell lines and animal models with results from human islets, but then it is important to consider the limitations of cross‐species comparisons, as miRNA targets are not always conserved, even when the miRNAs themselves are. Consequently, caution in the interpretation of results is essential and key experiments should, whenever possible, be validated in human islets.

## 4 Extracellular Vesicle‐Mediated miRNA Communication

MiRNA regulation has mainly been investigated as a local process within the miRNA‐producing cell, but several publications have demonstrated miRNAs in the circulation both bound to carrier molecules and contained in extracellular vesicles (EVs). EVs are generally believed to play an important role in intercellular communication and, interestingly, there is emerging evidence that EV‐mediated communication may play a role in T2D development [77]. Beyond secretory granules, β‐cells also secrete multivesicular bodies and EVs, and their dysregulated release in T2D correlates with reduced granule density and altered spatial organization, suggesting that EV pathways may intersect with exocytosis and contribute to disease‐associated signaling defects within the islet and systemically [78].

### 4.1 Circulating miRNAs as Biomarkers in Diabetes

Extensive research is focused on the possibility of using circulating miRNAs as biomarkers for early detection and diagnosis of disease. For this purpose, miRNAs can be measured from purified EVs or directly from plasma, serum, or other bodily fluids. In the latter cases, both miRNAs in EVs and those bound to protein or protein complexes are captured. One of the first examples of how miRNAs can be used as biomarkers was published by Lawrie et al. [79] who showed that a panel of three miRNAs (miR‐21, miR‐155, and miR‐210) could be used to detect a specific type of lymphoma. Since then, the topic has drawn attention also within the diabetes field, and several miRNAs/miRNA panels have been suggested as biomarkers for T2D prediction ([80, 81] and Table 1).

**TABLE 1:** Published work describing extracellular vesicles taken up or released from insulin‐secreting cells.

References	EV origin	Recipient cell	MicroRNA	Major outcome
Katayama et al. (2025) [82]	Human islets	Human myotubes	miR‐136‐3p	Glucose uptake ↑ mitochondrial function ↑
Wang et al. (2025) [52]	Plasma from people with obesity +/− T2D, and db/db mice	MIN6 cells mouse and human islets	miR‐146b	Proliferation↓ apoptosis↑ insulin content↓ GSIS↓
Fan and Li (2025) [83]	Adipose tissue from obese mice, palmitate‐treated 3T3‐L1 cells	MIN6 cells	miR‐138‐5p	Insulin production↓ cell viability ↓
Yang et al. (2025) [84]	MIN6 cells	C2C12 cells	miR‐204‐5p	Insulin resistance↑
Zhang et al. (2024) [85]	Mouse adipocytes	Mouse beta‐cells	miR‐27a‐5p	Insulin secretion↑
Yu et al. (2024) [86]	RIN‐m5F cells	RIN‐m5F cells	Small RNA sequencing	Insulin secretion↑ (EV only)
Lee et al. (2023) [87]	Hypoxic mouse pancreatic stellate cells	Mouse islets	miR‐23a‐3p	Cell death ↑ beta cell apoptosis ↑
Zhu et al. (2022) [88]	Mouse pancreatic stellate cell lines	MIN6 cells	miR‐140‐3p and miR‐143‐3p	Apoptosis↑
Gao et al. (2021) [89]	Adipose tissue macrophages from obese mice	Mouse beta cells, MIN6 cells	miR‐155	Insulin secretion↓ insulin production↓ proliferation↑
Qian et al. (2021) [90]	Macrophages from HFD mice	MIN6 cells mouse islets	miR‐212‐5p	Insulin secretion↓
Li et al. (2021) [91]	FFA‐treated mouse islets and MIN6‐cells	Mouse hepatocytes	miR‐29a/b/c	Hepatic insulin signaling ↓
Xu et al. (2020) [92]	Mouse islets MIN6 cells, mouse and human serum	Mouse hepatocytes adipose tissue	miR‐26a	Insulin sensitivity↑
Fu et al. (2019) [93]	Hepatocytes from obese mice	MIN6 cells	miR‐7218‐5p	Proliferation↑
Shen et al. (2019) [94]	MIN6 cells	MS1 cells	miR‐127	Cell migration↑ tube formation↑
Fu et al. (2018) [95]	Cytokine‐ or streptozotocin‐treated mouse islets Serum from people with T1D and T2D, respectively.		miR‐375‐3p	Biomarker for islet damage
Zhang et al. (2018) [96]	Mouse islets MIN6 cells	Mouse liver and skeletal muscle	miR‐223	GLUT4↑
Guay et al. (2015) [97]	MIN6‐B1 cells +/− cytokines, INS‐1832/13 cells, mouse, rat, and human islets	MIN6‐B1 cells mouse islet cells		Apoptosis ↑ (EV only)
Wan et al. (2017) [98]	Serum from people with T2D		miR‐7	Biomarker for T2D
Tsukita et al. (2017) [99]	Serum from bone marrow transplanted mice, mice bone marrow cells	+/− streptozotocin treated mouse islets	miR‐106b‐5p, miR‐222‐3p	Β‐cell regeneration and proliferation↑
Jalabert et al. (2016) [100]	Mouse myocytes C2C12 cells	MIN6B1 and 3 T3 cells	miR‐16 and more	Altered gene expression proliferation↑
Figliolini et al. (2014) [101]	Human islets	human islet endothelial cells	miR‐375, miR‐200c and miR‐21	Angiogenesis↑ anti‐apoptotic effects↑ cell migration↑ (EV only)

*Note:* The note “EV only” indicates that the article only describes how EVs impact the major outcome and not how specific miRNA(s) inside the EVs might be involved.

The biomarker field is rapidly developing, but urgently needs greater consistency. A recent meta‐analysis on available published data in T2D reports high variability among the results, which clearly hampers progress [102]. The reasons for the discrepancies between results are unclear. MiRNAs are stable in circulation and, importantly, not very sensitive to experimental processing and storage conditions [103], thus, other factors are probably more important contributors. The design of the study is critical. A comparative cross‐sectional study comparing circulating miRNAs in a group of people with T2D with a group without will only identify associations. A prospective study, on the other hand, can provide a temporally plausible association, even though causality is not fully proven, since it measures the miRNA expression at baseline and follows the individuals forward in time to see if they develop T2D.

In a study comparing serum from people with and without T2D, Wan et al. reported elevated circulating miR‐7 [98] and showed that most of this miRNA is not packaged within EVs but instead circulates freely bound to other molecular carriers. In a separate investigation, Gallo et al. evaluated baseline expression of specific miRNAs in healthy people who later developed diabetes and cardiovascular disease [80]. In this study, miR‐7e and miR‐483‐5p expression were significantly associated with incident diabetes after Bonferroni correction, and expression of additional miR‐7 variants and miR‐126 showed nominal positive associations. At least one other prospective study has presented a relationship between circulating miR‐126 and incident diabetes [81]. Recently, plasma miRNAs have been proposed as biomarkers of impaired β‐cell function, with miR‐34a‐5p, miR‐1306‐p, and miR‐335‐5p linked to early dysfunction [28]. Notably, miR‐335 has been shown to regulate β‐cell exocytosis [104].

We recently examined dynamic changes in circulating miRNA expression during an oral glucose tolerance test (OGTT) [50]. We reported that some miRNAs, such as miR‐122‐5p and miR‐34a‐5p, remained stable throughout the test, whereas others, including miR‐223‐3p, exhibited substantial fluctuation, and that this variation changed direction in people with diabetes. All three miRNAs were identified as potential biomarkers for cystic fibrosis‐related diabetes (CFRD). This work highlights the value of assessing acute miRNA responses to metabolic challenges and suggests that conducting a similar study focused on T2D could provide important new insights into miRNA regulation during diabetes development.

### 4.2 miRNAs in Intra‐Islet and Inter‐Organ Crosstalk

Most studies have examined the effects of EVs on insulin‐sensitive target tissues, but effects on insulin secretion have also been reported [105]. To evaluate the current knowledge on islet‐related EVs, Table 1 was created using the search terms (microRNA) AND ((exosome) OR (extracellular vesicle)) AND (islet) in PubMed in January 2026. The search resulted in a list of 88 articles. The abstracts of these articles were screened, and only original research articles clearly addressing the search terms stated above, containing experimental data, and focused on T2D were considered for the table. Jalabert et al. showed that EVs isolated from muscles from mice fed a HFD display increased levels of miR‐16 expression. These EVs were taken up by β‐cells, providing proof of concept that miRNA delivery from circulating EVs can affect β‐cell physiology [100]. Another direct connection between T2D and circulating miRNAs was provided by Zhang et al., who showed that increased levels of miR‐27a‐5p in islets from obese mice with a concomitant reduction in insulin secretion were explained by miR‐27a‐5p delivered from visceral adipose tissue via EVs rather than *de novo* synthesis in the β‐cells [85]. Once inside β‐cells, miR‐27a‐5p suppressed the Ca^2+^ channel gene *Cacna1c*, thereby reducing glucose‐stimulated insulin secretion. Blocking miR‐27a‐5p in adipose tissue or its vesicles improved β‐cell function and glucose tolerance in diabetic mice. Also, M1 macrophages can, under stressful circumstances, release miR‐212‐5p‐containing EVs that enter β‐cells and hamper insulin secretion via miR‐212‐5p mediated mechanisms [90].

EVs are also released from β‐cells and can impact other metabolically active tissues. For instance, a study by Yang et al. published last year [84] demonstrated that miR‐204‐5p levels increased in obesity and contributed to insulin resistance in skeletal muscle. The authors showed that islet β‐cells exposed to elevated fatty acids secreted exosomes enriched in miR‐204‐5p, which were taken up by muscle cells and impaired insulin signaling through suppression of *Sirt1*. Gain‐ and loss‐of‐function experiments confirmed that miR‐204‐5p reduced glucose uptake and disrupted the AKT/GLUT4 pathway in muscle, while inhibition of miR‐204‐5p restored insulin sensitivity. These results complement the work of Shalev and colleagues, who previously showed that miR‐204 regulates β‐cell function by repressing insulin transcription [106]. Hence, the role of miR‐204 extends beyond β‐cells to peripheral tissues, emphasizing its broader metabolic impact within diabetes pathophysiology. Human data on EV‐mediated miRNA signaling are still limited, as most studies so far have relied on cell lines and mouse models (Table 1). Nevertheless, work in β‐cell lines demonstrates that EVs can transfer miRNAs between islet cells [97], adding a layer of communication that complements hormonal, neurotransmitter, and other established signaling pathways.

The investigation of EV‐mediated intercellular communication is hindered by the pronounced heterogeneity of EV populations, which differ in size, origin, cargo, and function, complicating both their isolation and functional characterization. In addition, current isolation and enrichment methods (ultracentrifugation, density gradient centrifugation, ultrafiltration and size‐exclusion chromatography) lack standardization. This often yields preparations with varying purity and limited comparability across studies [107]. Moreover, the absence of robust methods to quantify total EV yield and distinguish functionally relevant subpopulations complicates the assessment of their role in cell–cell crosstalk. Nevertheless, ongoing advances in isolation technologies, single‐vesicle analysis, and standardization efforts are expected to improve resolution and reproducibility, thereby enabling a more precise understanding of EV‐mediated communication in health and disease. Collectively, these findings highlight a rapidly expanding field in which miRNAs facilitate cross‐tissue and cross‐cell communication.

### 4.3 Therapeutic Potential of Circulating miRNAs and EVs as Delivery Agents in T2D

Injection of miRNA mimics or antisense inhibitors into the circulation highlights the potential of miRNAs as therapeutic targets. In our studies, we administered antagomirs against miR‐132 in mice [108], resulting in reduced miR‐132 expression in islets, lower blood glucose, and increased insulin secretion. We also used locked nucleic acid (LNA) inhibitors targeting miR‐200c in islets from people with T2D, which improved insulin secretion [71].

However, the path to clinical application remains long. One challenge arises from the pleiotropic nature of miRNAs, which regulate multiple pathways within complex networks. It will therefore be important to identify key miRNAs that coordinate the regulation of pathways in the same direction, either promoting or impairing β‐cell survival and function. Targeting such miRNAs, either through inhibition with antagomirs or enhancement using miRNA mimics, may enable the simultaneous modulation of multiple pathways in a therapeutically beneficial manner.

Although several candidates have entered early‐phase trials, none have yet been approved. One of the first examples [109], RG‐125/AZD4076 targeting miR‐103/107 in the liver improved glucose homeostasis in preclinical models and advanced to clinical evaluation, providing proof of concept. Advances in oligonucleotide chemistry have enhanced stability, delivery, and specificity [110]. This facilitates clinical translation and supports the future development of antagomirs as potential therapies to improve insulin secretion in T2D.

Recently, therapeutics focus has also shifted toward EVs as promising delivery platforms [111, 112] due to their low immunogenicity and intrinsic ability to mediate intercellular communication and cargo transport. Their therapeutic potential is further enhanced by engineering both their cargo and surface properties, enabling targeted delivery of miRNAs, RNA therapeutics, gene‐editing tools, and other bioactive molecules. Compared with synthetic systems such as lipid nanoparticles, EVs offer advantages in biocompatibility and efficient interaction with recipient cells, although important challenges remain, including scalable production, controlled cargo loading, and tissue‐specific targeting. Despite these limitations, rapid advances in EV engineering and biology are accelerating their development toward clinical applications, positioning EVs as a highly promising platform for future precision therapies targeting insulin secretion and T2D.

## 5 Future Perspectives and Translational Outlook

The coming years are likely to bring substantial advances in our understanding of miRNAs as regulators of diabetes pathophysiology. Growing evidence highlights the importance of miRNA‐mediated islet and inter‐organ communication, particularly via EVs, which is likely to redefine how we conceptualize metabolic crosstalk in both health and disease. At the same time, the identification of miRNAs that directly influence the stimulus‐secretion coupling and the exocytotic machinery of β‐cells offers new therapeutic opportunities to target early β‐cell dysfunction in T2D.

Continued development of computational pipelines and integrative bioinformatic frameworks will further enable the discovery of complex miRNA–mRNA–protein networks and help clarify causal relationships. Methodological innovations are also on the horizon. Although single‐cell small RNA‐sequencing remains technically challenging, advances in bulk purification of α‐ and β‐cells combined with high‐resolution proteomics may generate more comprehensive molecular maps of miRNA–mRNA networks in islet cells. Together, these developments position miRNAs as promising candidates for both therapeutic manipulations through miRNA mimics, inhibitors, or EV‐based delivery systems, and for biomarker development, where disease‐associated miRNA signatures may enhance early detection, risk stratification, and treatment monitoring in diabetes.

## Author Contributions

**Mototsugu Nagao:** writing – review and editing. **Alexandros Karagiannopoulos:** writing – review and editing, data curation. **Lena Eliasson:** conceptualization, writing – original draft, writing – review and editing, project administration, resources, visualization, funding acquisition. **Anna Wendt:** writing – original draft, writing – review and editing, visualization, data curation.

## Funding

This work was supported by the Swedish Foundation for Strategic Research (IRC15‐0067), Vetenskapsrådet (2009‐1039, 2024‐03597), Diabetesfonden (DIA2025‐978), Diabetes Wellness Sweden (PG‐2025‐038), Barndiabetesfonden.

## Conflicts of Interest

The authors declare no conflicts of interest.

## Data Availability

Data sharing not applicable to this article as no datasets were generated or analysed during the current study.
